# Prognostic value of chemotherapy response score in advanced ovarian cancer: a single-center retrospective analysis

**DOI:** 10.1590/1516-3180.2025.2881.15082025

**Published:** 2025-11-03

**Authors:** Hamdullah Sözen, Yagmur Minareci, Atahan Toyran, Ibrahim Yalçin, Semen Önder, Aysel Bayram, Sidar Bağbudar, Mustafa Albayrak, Müge Ateş Tikiz, Pınar Mualla Saip, Samet Topuz, Mehmet Yavuz Salihoglu

**Affiliations:** I Associate Professor, Department of Gynecology and Obstetrics, Division of Gynecologic Oncology, Faculty of Medicine, Istanbul University, Istanbul, Türkiye.; II Assistant Professor, Department of Gynecology and Obstetrics, Division of Gynecologic Oncology, Faculty of Medicine, Istanbul University, Istanbul, Türkiye.; III Fellow, Department of Gynecology and Obstetrics, Division of Gynecologic Oncology, Faculty of Medicine, Istanbul University, Istanbul, Türkiye.; IV Associate Professor, Department of Gynecology and Obstetrics, Division of Gynecologic Oncology, Faculty of Medicine, Dokuz Eylül University, Izmir, Türkiye.; V Full Professor, Department of Pathology, Faculty of Medicine, Istanbul University, Istanbul, Türkiye.; VI Assistant Professor, Department of Pathology, Faculty of Medicine, Istanbul University, Istanbul, Türkiye.; VII Assistant Professor, Department of Pathology, Faculty of Medicine, Istanbul University, Istanbul, Türkiye.; VIII Associate Professor, Department of Gynecology and Obstetrics, Division of Gynecologic Oncology, Faculty of Medicine, Istanbul University, Istanbul, Türkiye.; IX Fellow, Department of Gynecology and Obstetrics, Division of Gynecologic Oncology, Faculty of Medicine, Akdeniz University, Antalya, Türkiye.; X Professor, Institute of Oncology, Department of Medical Oncology, Istanbul University, Istanbul, Türkiye.; XI Full Professor, Department of Gynecology and Obstetrics, Division of Gynecologic Oncology, Faculty of Medicine, Istanbul University, Istanbul, Türkiye.; XII Full Professor, Department of Gynecology and Obstetrics, Division of Gynecologic Oncology, Faculty of Medicine, Istanbul University, Istanbul, Türkiye.

**Keywords:** Ovarian neoplasms, Neoadjuvant therapy, Survival analysis, Interval debulking surgery, Chemotherapy response score, High-grade serous ovarian carcinoma, Overall survival, Disease-free survival

## Abstract

**BACKGROUND:**

The chemotherapy response score (CRS) is a histopathological tool used to assess the tumor response in patients with high-grade serous ovarian carcinoma (HGSC) undergoing neoadjuvant chemotherapy (NACT) followed by interval debulking surgery (IDS).

**DESIGN AND SETTING:**

This single-center retrospective study was conducted at the Faculty of Medicine at Istanbul University. The study included patients treated between January 1, 2010, and December 31, 2017 at a tertiary care hospital specializing in gynecologic oncology.

**OBJECTIVES:**

This study aimed to evaluate the prognostic significance of omental and adnexal CRS in predicting overall survival (OS) and disease-free survival (DFS) in patients with advanced HGSC undergoing NACT followed by IDS.

**METHODS:**

Data from 79 patients with advanced HGSC treated with NACT followed by IDS between 2010 and 2017 were analyzed. CRS was applied to both omental and adnexal samples, and its association with OS and DFS was evaluated. Statistical analyses were performed using univariate and multivariate methods with a significance level of P < 0.05.

**RESULTS:**

Omental CRS 1-2 was identified as an independent predictor of decreased OS (hazard ratio 2.69; 95% confidence interval 1.26–5.76, P = 0.010), whereas adnexal CRS 1-2 did not significantly impact DFS or OS in multivariate analysis. Patients with omental CRS 3 had superior outcomes, with a 5-year OS rate of 72%, compared to 30.8% in the CRS 1–2 group. The median DFS of the CRS 1–2 group was 19 months, whereas that of the CRS 3 group was 35 months (P = 0.005).

**CONCLUSIONS:**

Omental CRS is a strong independent predictor of OS in patients with advanced HGSC, whereas adnexal CRS has limited prognostic value. CRS should be considered in clinical practice to guide treatment decisions, and further research is warranted to refine its use by using molecular and radiological markers.

## INTRODUCTION

 Although not the most common, ovarian cancer is the deadliest gynecological malignancy. The World Health Organization reports that approximately 225,500 new cases are diagnosed annually, and 140,200 deaths occur, making it the seventh most prevalent and eighth leading cause of cancer-related deaths among women globally.^1,2^ Approximately 80% of cases are diagnosed at an advanced stage, with high-grade serous carcinoma (HGSC) being the most common histological type.^3,4^

 Surgery, often combined with chemotherapy, either as neoadjuvant chemotherapy (NACT) followed by interval debulking surgery (IDS) or primary debulking surgery (PDS) followed by adjuvant chemotherapy, remains the cornerstone of treatment for advanced-stage ovarian cancer. The use of NACT and IDS has increased in recent years because of favorable results from two randomized phase III trials, which showed similar disease-free survival (DFS) and overall survival (OS) with lower surgical morbidity and mortality rates compared to those undergoing PDS.^5,6^

 Another advantage of NACT followed by IDS is that it can provide an opportunity for future prognostic risk assessment through histopathological evaluation of the tumor response to chemotherapy. Böhm et al.^7^ developed the chemotherapy response score (CRS), which is a simple and reproducible scoring system based on post-therapy evaluation of the tumor architecture and microenvironment at the omental site. They demonstrated that CRS was significantly correlated with DFS, whereas the results for OS were mixed. 

 Böhm et al.^7^ proposed a simplified three-tier system: CRS 1, minimal tumor response; CRS 2, moderate tumor response with easily identifiable residual neoplastic foci; and CRS 3, complete or near-complete response with no residual neoplastic cells or minimal irregularly scattered tumor cells up to 2 mm in maximum size. This three-tiered scoring system demonstrated a significant prognostic difference between the CRS 1–2 and CRS 3 groups and improved interobserver reproducibility. Consequently, the three-tiered CRS has been incorporated into the International Collaboration on Cancer Reporting (ICCR) and College of American Pathologists (CAP) guidelines for the histopathologic reporting of ovarian carcinoma.^7,8^ Currently, the ESGO-ESMO-ESP consensus conference recommendations on ovarian cancer state that CRS performed during IDS on an omental (preferred) or adnexal specimen provides valuable prognostic information and is thus recommended.^9^

## OBJECTIVE

 This study aimed to validate the prognostic role of the CRS system in a cohort of patients from a clinic that has been an ESGO-accredited advanced ovarian cancer surgery center since 2018. 

## METHODS

### Study design

 The study protocol was reviewed and approved by the Istanbul Faculty of Medicine Clinical Research Ethics Committee at Istanbul University (approval number: 2024/2413). All the patients provided informed consent for the use of their medical information upon admission. All surgeries were performed by gynecologic oncologists. 

 This study was a retrospective analysis, and the sample size was determined based on the available data from patients who met the inclusion criteria within the defined study period (January 1, 2010, to December 31, 2017). No prospective sample size calculations were performed. The retrospective nature of the study allowed us to include all eligible patients, which improved the generalizability of the findings to this patient population. 

### Study population

**Figure 1:**
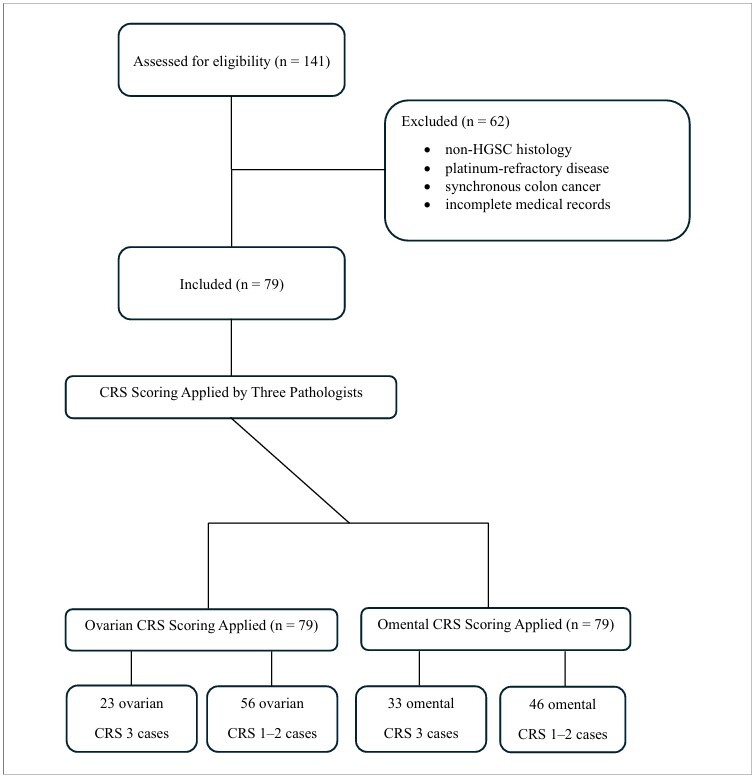
Flow diagram of the study.

 This study reviewed women with a postoperative histopathological diagnosis of advanced HGSC, treated between January 1, 2010, and December 31, 2017. Selection for NACT at our center was determined by a multidisciplinary tumor board after standardized preoperative staging using whole-abdomen magnetic resonance imaging and fluorine-18 fluorodeoxyglucose positron emission tomography/computed tomography. NACT was favored when complete or optimal cytoreduction was deemed unlikely based on objective imaging findings, including involvement or encasement of the superior mesenteric artery; multiple hepatic parenchymal metastases; involvement of the pancreatic head or body; infiltration of the hepatic hilum; extra-abdominal metastases (cerebral, pulmonary, osseous, or diffuse pleural carcinomatosis); or diffuse miliary carcinomatosis of the small-bowel serosa where resection would be expected to risk short-bowel syndrome. Patient-related factors also prompted NACT when medically unfit status was present, particularly Eastern Cooperative Oncology Group (ECOG) performance status ≥ 2 or significant comorbidities precluding extensive cytoreduction. Surgical pathology reports and/or clinical records were reviewed for each case to identify patients who received neoadjuvant platinum-based chemotherapy with carboplatin and paclitaxel. In this cohort, 141 women underwent NACT before undergoing IDS. Because the use of CRS has been validated and recommended only for HGSC, all other epithelial ovarian cancer histotypes were initially excluded from the present study. Patients with synchronous malignancies, incomplete clinicopathological records, and those who did not undergo optimal cytoreductive surgery were excluded. Women with platinum-refractory diseases were excluded from the study. The study sequence is illustrated in Figure 1. 

 Germline or somatic BRCA 1/2 status was not routinely obtained during the study period because of local reimbursement policies; therefore, genetic data were not systematically collected and were not included in the analyses. 

### Pathology review

 The ICCR issued guidelines on reporting serous ovarian cancer, advising the inclusion of a 3-point scoring system described by Böhm et al.^7^ as the standard for the assessment of tumor regression following NACT. 

 All available hematoxylin and eosin stained slides from the surgical specimens that were diagnosed as HGSC in the Pathology Department of Istanbul Faculty of Medicine were re-evaluated by three pathologists (S.Ö, A.B, S.B), including an expert gynecologic pathologist (S.Ö). Each slide representing the omentum and adnexa was scored independently by pathologists according to a three-tier system. The summary of the scoring system proposed by Böhm et al. is as follows: Score 1: No or minimal tumor response (mainly viable tumor with no or minimal regression-associated fibroinflammatory changes, limited to a few foci); Score 2: Partial tumor response (both tumor groups and regression-associated fibroinflammatory changes are easily identifiable); Score 3: Complete or near-complete response (mainly regression, with few irregularly scattered individual tumor cells or cell groups, all measuring < 2 mm, or no residual tumor identified).^7^ The cases that did not receive the same score from all three pathologists were evaluated together, and a consensus was established for outcome analysis. 

### Statistical analysis

 Patient data, including age, disease stage, histology, CA-125 levels at diagnosis and completion of NACT, and residual tumors after IDS, were collected from computerized medical records. The level of CA-125 reduction attributable to NACT was defined as the difference between the highest pretreatment and pre-surgical CA-125 measurements. The survival status of patients was determined as alive or dead at the time of the last follow-up. This was confirmed by performing a Social Security Death Index search for all study participants with recorded deaths. After initial diagnosis, recurrence was defined as documentation of metastasis with serum CA-125 measurement and imaging techniques after a DFS ≥ 3 months. DFS was defined as the time from surgery to the first identification of recurrence by radiological imaging and serum CA-125 measurement or death from any cause, whichever occurred first, or the date of the last contact for patients who remained alive without recurrent disease. OS was calculated as the period between the initial diagnosis of HGSC and the date of death or last contact. The surviving patients were censored at the last follow-up. Survival analysis was based on the Kaplan–Meier method, and the results were compared using a log-rank test. The chi-square test and Student’s t-test for unpaired data were used for statistical analysis. Cox regression analysis was used to determine the factors affecting survival, presented as hazard ratios (HRs) and 95% confidence intervals (95% CI), unadjusted or adjusted for all factors. All variables with a p value < 0.05 in univariate analysis were included in the multivariate analysis. All statistical analyses were performed using SPSS software (version 23.0; SPSS Inc., Chicago, IL, USA). Statistical significance was set at P < 0.05. 

## RESULTS

 A total of 62 patients were excluded because of non-HGSC histology, platinum-refractory disease, synchronous colon cancer, or incomplete medical records. Table 1 shows a comparison of the baseline characteristics of the 79 patients included in this study. Twenty-three adnexal CRS 3 cases were compared with 56 adnexal CRS 1–2 cases. Age, menopausal status, pre-NACT CA-125 levels, post-NACT CA-125 levels, percentage of CA-125 drop, stage, presence of ascites at surgery, number of NACT cycles, and recurrence rates were similar between the groups. The time to chemotherapy after IDS (47 days vs. 30 days, respectively) and median follow-up (72 months versus 48.50 months, respectively) were significantly longer in adnexal CRS 3 cases than in adnexal CRS 1–2 cases. Patients with adnexal CRS 3 were more likely to have a high omental CRS than that of those with adnexal CRS 1–2 (69.6% and 30.4%, respectively). 

**Table 1:** Baseline characteristics of the study cohort.

/		**Adnexal CRS 1–2 (n = 56)**	**Adnexal CRS 3 (n = 23)**	**P**
Age, years (median)		60 (38–80)	58 (39–78)	0.927
Menopausal status				
	*Pre-menopause*	16 (28.6%)	4 (17.4%)	0.299
	*Post-menopause*	49 (71.4%)	19 (82.6%)	
Pre NACT CA 125 levels (median, IU/ml)		1192.50 (259–10858)	980 (270–8169)	0.553
Post NACT CA 125 levels (median, IU/ml)		32.50 (7–450)	29 (3–355)	0.524
Percent of CA 125 drop %, (median)		96.32 (71.76–99.53)	96.53 (81.08–99.86)	0.742
Stage				
	*Stage IIIC*	47 (83.9%)	17 (73.9%)	0.35
	*Stage IV*	9 (16.1%)	6 (16.1%)	
Presence of ascites at surgery, n				
	*Present*	23 (41.1%)	8 (34.8%)	0.603
	*Absent*	33 (58.9%)	15 (65.2%)	
NACT cycles				
	*3–4*	23 (41.1%)	10 (43.5%)	0.844
	*> 4*	33 (58.9%)	13 (56.5%)	
Time to chemotherapy after interval debulking surgery, days (median)		30 (15-75)	47 (24-95)	**0.001**
Omental CRS				
	*Score 1–2*	39 (69.6%)	7 (30.4%)	**0.001**
	*Score 3*	17 (30.4%)	16 (69.6%)	
Recurrence, N				
	*Present*	43 (76.8%)	16 (69.6%)	0.503
	*Absent*	13 (23.2%)	7 (30.4%)	
Median follow up, months		48.50 (15–104)	72 (24–129)	0.014

CA 125, cancer antigen 125; NACT, neoadjuvant chemotherapy; CRS, chemotherapy response score.

 For the entire cohort, univariate analysis revealed that adnexa CRS 1-2 (P = 0.016) and Omental CRS 1–2 (P = 0.007) were significant predictors of decreased DFS. Multivariate analysis showed that none was determined to be an independent risk factor for decreased DFS (Table 2). 

**Table 2:** Univariate and multivariate analyses for prognostic factors for disease-free survival in the entire cohort.

**Variable**	**Univariate analysis HR 95% CI P**	**Multivariate analysis HR 95% CI P**
Age	0.912	
Menopausal status (post-menopause vs. pre-menopause)	0.73	
Pre NACT CA 125 levels	0.443	
Post NACT CA 125 levels	0.564	
Percent of CA 125 drop	0.878	
Stage (IV vs. IIIC)	0.69	
Ascites (present vs. absent)	0.168	0.755
NACT cycles (> 4 vs. 3–4)	0.059	0.074
Time to chemotherapy after interval debulking surgery	0.079	0.46
Adnexa CRS (1–2 vs. 3)	**2.10 1.14–3.86 0.016**	0.096
Omental CRS (1–2 vs. 3)	**2.08 1.22–3.54 0.007**	0.112

HR, hazard ratio; CI, confidence interval; LN, lymph node.

 In the entire cohort, univariate analysis revealed that the presence of ascites (P = 0.002), adnexa CRS 1–2 (P = 0.011), and omental CRS 1–2 (P < 0.001) were significant factors for decreased OS. At the end of multivariate analysis, only omental CRS 1–2 (HR 2.69; 95% CI 1.26–5.76, P = 0.010) was identified as independent predictor of decreased OS as shown in Table 3. 

**Table 3:** Univariate and multivariate analyses for prognostic factors for overall survival in the entire cohort.

**Variable**	**Univariate analysis HR 95% CI P**	**Multivariate analysis HR 95% CI P**
Age	0.992	
Menopausal status (post-menopause vs. pre-menopause)	0.756	
Pre NACT CA 125 levels	0.423	
Post NACT CA 125 levels	0.675	
Percent of CA 125 drop	0.363	
Stage (IV vs. IIIC)	0.69	
Ascites (present vs. absent)	**2.59 1.43–4.70 0.002**	1.86 1–3.48 0.05
NACT cycles (> 4 vs. 3–4)	0.153	0.198
Time to chemotherapy after interval debulking surgery	0.213	0.838
Adnexa CRS (1–2 vs. 3)	**2.62 1.24–5.53 0.011**	0.182
Omental CRS (1–2 vs. 3)	**3.56 1.77–7.16 < 0.001**	**2.69 1.26–5.76 0.01**

HR, hazard ratio; CI, confidence interval; LN, lymph node.

**Figure 2:**
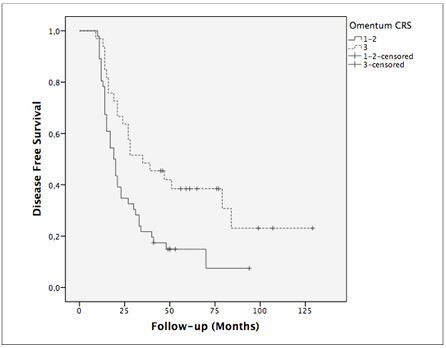
Kaplan–Meier curve showing disease-free survival based on omental CRS (1–2 versus 3).

**Figure 3:**
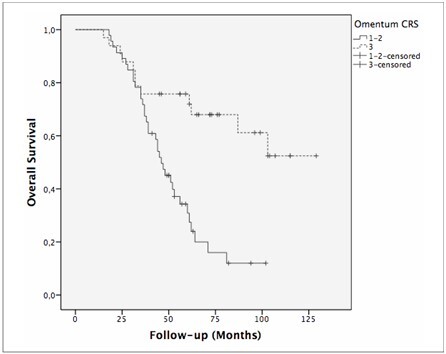
Kaplan–Meier curve showing overall survival based on omental CRS (1–2 versus 3).

 Because only omental CRS was identified as an independent predictor of decreased OS, log-rank analysis was performed to assess the impact of omental CRS on survival. The median DFS for women with omental CRS 1–2 was 19 months (95% CI 14.8–23.1, standard error [SE] 2.11) compared to 35 months (95% CI 13.1–56.8, SE 11.14) in the omental CRS 3 group (P = 0.005) (Figure 2). The 5-year DFS rate was higher in the omental CRS 3 group (38.5% in the omental CRS 3 group vs. 14.9% in the omental CRS 1–2 group). The median OS of the omental CRS 1–2 group was 46 months (95% CI 38.8–53.1, SE 3.67), while the median OS of the omental CRS 3 group was not yet reached (P < 0.001) (Figure 3). When the 5-year OS rates were examined; superior outcomes were observed in omental CRS 3 cases than in omental CRS 1–2 cases (72% versus 30.8%, respectively). 

 At the time of reporting, of the 33 women in the omental CRS 3 group, 12 (36,4%) had died, whereas 21 (63,6%) were alive. The corresponding figures were 35 (76,1%) and 11 (23,9%) in omental CRS Groups 1 and 2, respectively. 

## DISCUSSION

 In this study, we investigated the prognostic significance of CRS in patients with advanced-stage HGSC, with a particular focus on the differences in outcomes between adnexal and omental CRS. Our results demonstrated that omental CRS 1–2 was a significant independent predictor of decreased OS, whereas neither adnexal CRS 1–2 nor omental CRS 1–2 were independent predictors of decreased DFS in multivariate analysis. These findings reinforce the importance of the omental CRS as a critical site for assessing chemotherapy response in HGSC. 

 The CRS system, initially developed and validated by Böhm et al.,^7^ has been accepted as a reliable and reproducible marker for evaluating histopathologic response to NACT.^7^ Although the importance of the CRS score is emphasized in the ESGOESMO-ESP consensus conference recommendations on ovarian cancer, the relationship between the CRS score and DFS and OS outcomes is inconsistent in studies in the literature (Table 4).^7,9-18^ Our findings align with previous studies that established the prognostic value of CRS, particularly in the omentum, where higher CRS 3 is associated with significantly improved OS compared to that with CRS 1–2.^14-16,19^ In line with the work of Santoro et al., we found that omental CRS 3 patients had superior survival outcomes, with a 5-year OS rate of 72%, compared to 30.8% in omental CRS 1–2 patients.^14^

**Table 4:** Summary of studies evaluating CRS in relation to DFS and OS.

**Study**	**Sample Size**	**Scoring System**	**Period of Enrollment**	**Results**	**HR, P**
Böhm et al.^7^	62 (TC)	Six-tier omental and adnexal	2009–2014	A significantly improved DFS for mCRS 4–5 compared with mCRS 2–3 (Adjusting for age, stage, and residual disease) A significant OS benefit for mCRS 4–5	mCRS 2–3 vs. mCRS 4–5: Median survival, 11.3 vs. 32.1 months; aHR, 6.13; 95% CI, 2.13–17.68; P < 0.001 aHR, 24.97; 95% CI, 2.35–265.6; P < 0.01 P = 0.59 P = 0.12
	71 (VC)	Three-tier omental	1999–2012	Adnexal scores not associated with DFS or OS Improved DFS for mCRS3 compared with mCRS 1–2 Nonsignificant trend for OS	mCRS 1–2 vs. mCRS 3: median survival, 12 vs. 18 months; aHR, 3.60; 95% CI, 1.69–7.66; P < 0.001 mCRS 1–2 vs. 3: median survival, 28.4 vs. 45.1 months; aHR, 1.81; 95% CI, 0.79–4.14; P = 0.15
Lee et al.^10^	110	Three-tier omental and adnexal	2006–2014	İmproved DFS for mCRS3 compared with mCRS 1–2 No significant difference in OS between mCRS 1–2 and mCRS 3 Adnexal CRS showed no significant association with outcome	The median DFS of CRS 1–2 vs. CRS 3 14.5 vs. 18.6 months, P = 0.016 P = 0.902 P = 0.317
Coghlan et al.^11^	71	Three-tier omental	2010–2014	İmproved DFS for mCRS3 compared with mCRS 1–2 No significant association for OS	HR, 2; 95% CI, 1.06–3.78; P = 0.032; median DFS, 26 months (mCRS 3) vs 16 months (mCRS 1–2) mCRS 1–2 vs. 3: HR, 1.57; 95% CI, 0.68–3.65; P = 0.291
Ditzel et al.^12^	68 (omental n = 65) (adnexal n = 59)	Three-tier omental and adnexal	2005–2012	İmproved DFS for AT mCRS3 compared with AT mCRS 1–2 No significant association with OS between AT mCRS 1–2 and AT mCRS3 Adnexal CRS showed no significant association with DFS and OS	AT mCRS 1–2 vs. 3; HR, 0.526; 95% CI, 0.306–0.904; P = 0.020 Median DFS, 10.9 vs. 18.9 months AT mCRS 1–2 vs. 3; HR, 0.608; 95% CI, 0.319–1.16; P = 0.131 Median DFS, 39.4 vs. 53.6 months P = 0.062 P = 0.055
Michaan et al.^13^	132	Three-tier omental and adnexal	2009–2014	Significantly longer DFS for mCRS 3, with no significant OS difference A significantly longer DFS but not OS for patients with ovCRS 3 Significantly longer DFS for cCRS 3, with no significant OS difference	P < 0.01 Median DFS = 7.5, 12, and 17 months for ovCRS 1, 2, and 3, P = 0.012 P < 0.01
Santaro et al.^14^	161	Three-tier omental and adnexal	2014–2017	Worsened DFS with mCRS1 and ovCRS1-2, Compared with mCRS3 and ovCRS3 Worsened OS with mCRS1 compared with mCRS3	mCRS1 vs. mCRS3: HR, 2.17; 95% CI, 1.41–3.33; P = 0.0004 mCRS2 vs. mCRS3: HR, 2.34; 95% CI, 1.35–4.04; P = 0.002 ovCRS1 vs. ovCRS3: HR, 2.53; 95% CI, 1.50–4.24; P = 0.001 ovCRS2 vs. ovCRS3: HR, 1.90; 95% CI, 1.08–3.37; P = 0.03 mCRS1 vs. mCRS3: HR, 2.75; 95% CI, 1.29–5.86; P = 0.01
Böhm et al.^15^	80	Three-tier omental	2009–2015	İmproved DFS with mCRS3 compared with mCRS2 İmproved OS with mCRS3 compared with mCRS2	Median DFS 13 months (mCRS 2) and 27 months (mCRS 3); HR 0.39 (95% CI 0.21–0.7), P = 0.002 (Adjusted for age, stage, and debulking status) Median OS was 31 months (mCRS 2) and 66 months (mCRS 3); HR 0.17 (95% CI 0.07–0.44), P = 0.0002 (Adjusted for age, stage, and debulking status)
	Zorzato et al.^16^	108	Three-tier omental	2007–2017	İmproved DFS with mCRS3 compared with mCRS1 İmproved OS with mCRS3 compared with mCRS1	HR 0.35; 95% CI, 0.2–0.61; P < 0.0001 HR 0.38; 95% CI, 0.18–0.82; P = 0.013
Lawson et al.^17^		158	Three-tier omental and adnexal	2013–2018	İmproved DFS with mCRS3 compared with mCRS 1–2 No association with OS between mCRS1-2 and mCRS3 İmproved DFS for ovCRS3 compared with ovCRS1-2 No association with OS between ovCRS1-2 and ovCRS3 İmproved DFS for cCRS3 compared with cCRS 1–2 No association with OS between cCRS 1–2 and cCRS3	HR 0.612, 95% CI: 0.378–0.989, P = 0.045 HR 0.96, 95% CI: 0.495–1.865, P = 0.91 HR 0.535, 95% CI: 0.297–0.963, P = 0.037 HR 0.734, 95% CI: 0.327–1.645, P = 0.45 HR 0.364, 95% CI: 0.148–0.896, P = 0.028 HR 0.66, 95% CI: 0.205–2.131, P = 0.49
	Liontos et al.^18^	48	Three-tier omental and adnexal	2011–2016	Median DFS was associated with CRS at omentum CRS at omentum was not associated with OS Adnexal scores not associated with DFS or OS	Median DFS was: 10.3 months (mCRS 1) (95% CI 7.4–15.7), 14 months (mCRS 2) (95% CI 12.2–22.9), 18.7 months (mCRS 3) (95% CI 13.5–31.3), P = 0.003 Median OS was: 29.3 months (mCRS 1) (95% CI 10.9-NR), 32 months (mCRS 2) (95% CI 16.4–46.9) 42.3 months (mCRS 3) (95% CI 30.8-NR), P = 0.182 P = 0.115 P = 0.428

DFS, disease-free survival; OS, overall survival; CRS, chemotherapy response score; mCRS, omental chemotherapy response score; ovCRS, ovarian chemotherapy response score; cCRS, combined chemotherapy response score; HR, hazard ratio; TC, test cohort; VC, validation cohort; AT, after traiing.

 One of the notable findings in our study was the absence of prognostic significance for adnexal CRS 1–2 in the multivariate analysis for DFS, which contrasts with some reports suggesting the relevance of adnexal CRS in predicting survival outcomes.^13,14,17,20^ This discrepancy may stem from inherent challenges in scoring adnexal disease, as previously noted in the literature. The adnexal site is often less reproducible, and adnexal CRS has shown variable prognostic significance across studies.^21^ Further research is needed to standardize the assessment of CRS in adnexal tissue and to determine its potential utility in guiding clinical decision-making.^19^

 Our study further underscores the critical role of the omental CRS in predicting long-term survival. As demonstrated in a meta-analysis by Cohen et al.,^19^ omental CRS 3 remains a strong independent predictor of both DFS and OS. In our cohort, the median OS for the omental CRS 1–2 group was 46 months (95% CI 38.8–53.1, SE 3.67), indicating a shorter survival compared to that of the omental CRS 3 group, for which the median OS was not yet reached. These results suggest that the extent of response to chemotherapy in the omentum can serve as a valuable surrogate for predicting treatment response and overall prognosis. 

 Despite the strengths of our study, including its well-characterized cohort and comprehensive follow-up data, some limitations must be acknowledged. The relatively small sample size may have limited the generalizability of our findings. Moreover, the exclusion of patients with platinum-refractory disease may have introduced selection bias, potentially underestimating the true prognostic significance of CRS. In addition, the absence of BRCA 1/2 data due to local reimbursement constraints during the study period precluded analysis of the potential influence of BRCA status on CRS distribution and survival outcomes. 

## CONCLUSION

 Our study highlights the prognostic value of omental CRS in advanced epithelial ovarian cancer, particularly for predicting OS. The use of CRS, especially in omental tissues, should be further explored as a clinical tool to guide therapeutic decision-making and follow-up strategies. Future research should focus on refining the CRS system and integrating it with molecular and radiological markers to enhance its prognostic utility. 

## Note:

The submitted version of this manuscript was posted on Research Square under DOI https://doi.org/10.21203/rs.3.rs-5425478/v1. The authors certify that this work is original, has not been published elsewhere and is not currently under consideration for publication elsewhere.
